# A case of prolonged sinus arrest for 5 minutes after Cryo‐balloon ablation to the left superior pulmonary vein

**DOI:** 10.1002/joa3.12268

**Published:** 2019-11-21

**Authors:** Tomonori Miki, Hirokazu Shiraishi, Takeshi Shirayama, Satoaki Matoba

**Affiliations:** ^1^ Department of Cardiovascular Medicine Kyoto Prefectural University of Medicine Kyoto Japan

**Keywords:** atrial fibrillation, cryoballoon ablation, pulmonary vein isolation, vagal response

## Abstract

A 63‐year‐old man was referred to our hospital for his palpitation due to atrial fibrillation. He was admitted for catheter ablation. Cryoablation was applied to the left superior pulmonary vein for 180 seconds, and its potential disappeared in 22 seconds. The lowest temperature was −45°C. Suddenly, sinus arrest was observed 1 minute after the completion of freezing. The right ventricle was paced but no atrial potential was observed for 5 minutes until normal sinus rhythm resumed. We report a case of severe sinus arrest after cryoablation to the left pulmonary vein.

## Graphical abstract

Sinus arrest was observed 1 minute after the cryo‐balloon ablation. No atrial potential was observed for 5 minutes until normal sinus rhythm resumed.

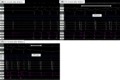

## 1 INTRODUCTION

Catheter ablation has become a novel treatment option for patients with symptomatic drug‐resistant atrial fibrillation (AF). Pulmonary vein isolation (PVI) becomes popular for AF ablation.^1^ A randomized trial showed that cryoballoon ablation (CBA) was noninferior to radiofrequency ablation (RFA).^2^ It is well known that RFA during PVI often induce vagal reflexes (VR). Similarly, during CBA, VR could result in sinus arrest and atrioventricular block.^3^

Although a report showed that bradycardia episodes by CBA due to VR lasted for about 1 minute at maximum,^3^, ^4^ we experienced a case of significant sinus arrest for about 5 minutes after CBA to the left superior pulmonary vein (LSPV).

## 2 CASE DESCRIPTION

A 63‐year‐old man was referred to our hospital for palpitation due to drug‐resistant paroxysmal AF. He had a history of faintness twice after drinking alcohol. He was admitted to our hospital for CBA. A CT image with contrast media showed no significant stenosis in the coronary artery and sinus node artery originating from the right coronary artery. A transesophageal echocardiography showed no thrombus. Ablation was performed under propofol‐induced moderate sedation. A 20 pole three site mapping catheter (BeeAT; Japan‐Life‐Line, Tokyo, Japan) was inserted into the coronary vein. A transseptal puncture was performed using an RF Needle (Japan‐Life‐Line) in an 8 F long sheath (SL0; AF Division, St. Jude Medical [SJM], Minneapolis, MN) under guide of the intracardiac echocardiography.

**Figure 1:**
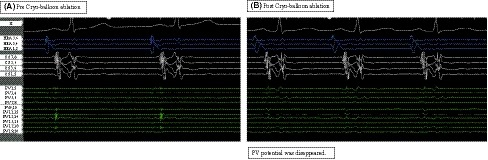
Pre‐ and postablation with Cryoballoon ablation. Cryoballoon ablation was applied to the left superior pulmonary vein, and the potential disappeared in 22 sec

After a transseptal puncture, Agilis sheath (SJM) was inserted to the left atrium. SL0 sheath was exchanged over a guidewire for a 15F steerable sheath (FlexCath Advance; Medtronic) and a 20 mm circular mapping catheter (Optima; SJM) was inserted via Agilis sheath for mapping PV potentials (Figure 1A). A spiral mapping catheter (Achieve; Medtronic) was used to advance a second‐generation CB (Arctic Front Advance; Medtronic) into PV. An electrode catheter for emergency backup pacing was placed to the right ventricle from the 10 F sheath.

**Figure 2:**
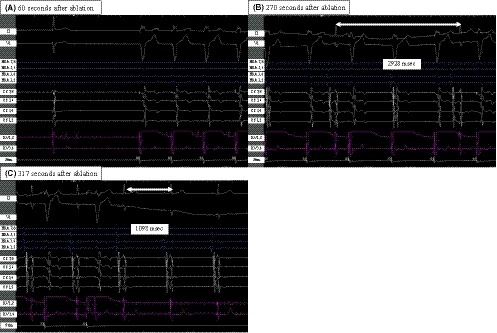
Sinus arrest after ablation. At the time of RV backup pacing, no atrial potential was observed. Atrial potential appeared gradually and returned to sinus rhythm after 5 min. Numbers in the figure show A‐A interval

A 28 mm CB was inflated proximal to the PV ostium, followed by a gentle push aiming for the complete sealing at the antral aspect of the PV. Freezing was started from the LSPV, and the potential disappeared in 22 seconds after the start of ablation (Figure 1B). Cryoablation was applied for 180 seconds. The lowest temperature was − 45°C. Suddenly, sinus rhythm disappeared 1 minute after CB deflation with systolic blood pressure (BP) drop to 42 mm Hg (Figure 2A) without obvious ST segment change. Patients came out in marked cold sweat without symptoms such as chest pain and nausea. We injected atropine sulfate 1 mg immediately, but systolic BP remained low at 67 mm Hg. Due to no improvement after 4 minutes follow‐up, a second dose of 1 mg atropine sulfate was injected. Right ventricular pacing was started but no atrial potential was observed. Sinus rhythm gradually appeared (Figure 2B), but it took 5 minutes until complete recovery (Figure 2C). After injection of atropine sulfate 2 mg, BP did not improve even after the sinus rhythm was recovered. Etilefrine 1 mg was injected and systolic BP was raised up to 120 mm Hg. The other PVs were isolated with CBA without complications. The electrophysiological study after PVI showed that maximum sinus node recovery time (SNRT) was normal with 1166 msec at 180 bpm. Wenckebach point of AV node conduction was 200 bpm. The effective refractory period (ERP) of the high right atrium was 200 msec with a basic pacing cycle length of 500 msec and the ERP of AV node conduction was less than 200 msec. There was no retrograde conduction. Otherwise there was no complication, and he was discharged as scheduled.

## 3 DISCUSSION

We report a case of significant sinus arrest occurred for about 5 minutes after CBA to the LSPV. It is well known that both RFA and CBA around PV ostia often induce VR.^4^

The incidence of VR was reported as high as 26.8%‐38.2%, and the median time of duration was 41‐50 seconds and the range was 10‐69.3 seconds.^3^, ^4^ Compared with such reports, the present case presented excessive activity of VR for a long time of 317 seconds before complete recovery.

As far as we know, there is no report that sinus arrest and bradycardia lasted for more than 5 minutes.

Holter monitoring and GP stimulation test were not performed. However, SNRT and ERP are both within normal range after procedure. Although these results may not reflect natural status due to post procedure, intrinsic sinus rhythm was intact. VR was reported to be related to epicardial adipose tissue (EAT) around the PV.^3^ A CT image in our case showed little EAT around left pulmonary vein (4.25 cm^3^).

Atropine is effective in preventing VR induced by CBA.^5^ For this patient, however, administration of atropine injection in two doses was not effective. Therefore, etilefrine was administered. We should have given atropine sulfate injection once more as indicated to follow ESC guidelines, but we did not try it due to low blood pressure. Vagal activity was too strong to be blocked by atropine injection. Transient bradycardia is often complicated with the ablation to this vein, provoking VR. There report showed that CBA to the right PV (RPV) markedly suppressed VR of LSPV.^4^

Patients with history of suspected neuroregulatory syncope may experience significant bradycardia during CBA. Therefore, we may need to avoid this response by CBA applying RPV first or by prophylactic atropine administration.

## 4 CONCLUSION

We report a case of severe sinus arrest after cryoablation of the left pulmonary vein.

## 5 CONFLICTS OF INTEREST STATEMENT

The authors declare that there is no conflict of interest.
